# Table of Meteorological Conditions in the City of Buffalo for December, 1854

**Published:** 1855-02

**Authors:** Sanford B. Hunt


					﻿ART. IX. — Record of Meteorological Conditions at Buffalo, for December
1854. By Sanford B. Hunt, M. D.
1^3 8
.	«p<	s
•	fe co -• 5	~
3	o	c-S fs 10	^3	_•
M	q q q o	S 8
Pi	2	- oo	g	-S	k	5	8
<1	4-(	CO	QQ	CO	Q	k—
I «	i’lti	I	i	ft'
■;	ofgs-j	g
s	*=30	68 fl	£	fl PC
_____________O	< << m	E-i___________tn	tn tn______________
•9jn;cjadtuax	S
JO OSu-BJ JSSJVOJg CT©«COOO©Tt<COOO>-ICi|'ctl©COinCOin-3j’COOOin'0<t-CT—ITj<—lOOCT—iCO jrf
■An pc uo m m m to in © o m m o co o m o o o co m o m o moaSoot-t—t-00 00
mmnn Train- •AOOCOCOaOaOCO-HCOCnOOrt'cl'ClOOCDi-t—<-tfCO-3CO—IOOrHClt--CC>ClClCO o
-ptmnH uBaivloa ooaiciQOciciaioociaociooooooaociciciaicicicioooioociaioooo 00 ci
k	T	„'d ~ciMtocoa>co«^ir-iOT4op®rti-cooo®inoio4 ct co ci ci -d 55
C	?uI°d CO TI? Cl. Cl CO CD 00 O CO -J' CT CT Tf —< t£> 00 CT CO CO 00 Cl irt —t 1.0	ct 00 o CO UO
5 -Ai9(j uBOftj odcdcdcit-iTf<'cdCTodidocdCT'dt2odcdCTocdr-i—ioidTjiidk-'ooo'id^ co
Q _______________CTCTCT	—	—ICT—<r-tCTCOCOCTCO	CO	CC Ci PA r. r-	CJ -C1CO CO	CO CO T —<	t—	CT	CT
■am™	CO	,.	CD	CO CO	CO	CO CO	CO CO	CO	CO	CO	It—
3 ,Inl “	t' CO .	CO	CO .	.	.	CO CO	. CO. CD	CO	.CO .CO. .	. . CO CO	. CD	CO .	tn	CO	03
-iaaaiaj uttapj o k o	ci i'O cc	?i P	-< ci t~2	ad	—< cd a id ci	ci m' -d cd	ci t-2	ci 06 fli ct	ci ci	t—'
_____________CO CT	CT	CT CT CT —I —<	CO COCOCTCOCO^COCT—I	pH CT Cl CO	Cj CO CO-+CT -<	CT	CT
I in o	uo	id o in ioioofloiooo>ooioinoin>o>oio	o’o m o	o	Tct
. •XlTnnnnTTl W S	CO	CO CO CO CO CO	CO CO CO CO CO CO CO CD COCO t-	CO CO CO CO	CO	CD CO O	CO	—I
u	j Cl OO	Cl	Cl 00 Cl Cl Cl	CO Cl 00 Cl 00 00 Cl 00 Cl ©OT Cl Cl Cl Cl	00	GO Cl ©	00	j©
8 •lUtOd' Maa!'0. ’W^'PCO.COCOCOCO.COCOCOCOCOCDCOCOCOQOCOCOCOCDCOCOCO	CO CT
c t.a- u. co t- —; an co ■’f id -d r- -d cot- co oi ct o' tr ct co co id co	'd -i
. ; G> ______ICT CT CT —ICT CT -3 —I COCOCTCTCOCOCOCTp-<_Ct—<■— COCOCOCOCO______________________Ct CT
.3Tm dma-r	S
2h 8Jn:*‘ ln8^ Mcoin’bcofficdNOKaiocioddriktN^mdirjTilHmadoo	ci oo
. ______co CT CTCTCT.CO I —‘TCCOCTCOCOCQpJiCOr-l—I	CT CT — CO CO COCO_____________________CT_CT
•	£ .m ..
o	3 m tnnfZ^xi aj^jjz; tn^Hntitri&i£i H
________________cd—tcocoTp-^r-ocoCTCT-dt'—iq—.CT-atootri’-^-tcocoCT^-^	ct  
g	spno[3j04jny|o O o o o o o o-< o o co o co o o o o o o o o o o o o o_o
m	‘.a ® 12 1 fl — “fl ‘fl o o in tedo — © oo o omUn"o'ia ci'nomIdo ooTt-
r . A!!P!LurlII cocococococococococococo—i—icacocDcocococococococo—icocococo—<ho
r 5_____________oiaocicioooicioooojiciooGoooooooooooci ciaocioociciooCici oo oo oo loo
&	"	iCl
g .][no T M3„ cocococococqcqcococococo co co co co to co co co co co co . co co co co . |ct
5	-i.	a.	(1	_; t— _'—■	oo cd t- td	cd	io co t- -d	-f	d	oi t-co'o	a5 «d -d CT	cd	id id co cd co	cd	id	red
.	tj,	_____CO Qj CO	CT	CT —I —	CT	CO CT CT CO	CO	-f<	CT — ■—	—	— CT —« CT	CO	CO CO CO -r -p	CT	CT	'CT
—	[jo
.	i-	•ajnj.duiax	-ji	cd	cd cd o o	cd	06 -I ct	id	ct od	cd	-i o' t-i t-i	cd	cd ct ci cd	id	ct	o
—_________co CO. CO	CT	CT CT CT Ot	CO	CO CO C0_ -f	-f	-r	CO CT —'	—<	O! CO — OJ	CO	CO Tf .co -C CT	CT	CO	I CO
<*' g-cg -d	• ...
= § g®^	<Z2 M 02 n n n n n n Jz<	n pq n ezi n ai cd tn
...................................................
___________< —I ifl -^t t- CO CO CT CT CT —I CO —< —I CT fl! —< Tf -C CO CO O ! CT CT —< —I —3 r-i —i
spnoojoxaiv k- © o o o t- m m o © co o oo ©(«5 cd q o os o q m © o o o o jo
iTStOLOiOiOLOLOLOOtQOtOOOOOiOiQtOtQiOtOtOOtOC^OO >O iTi CO
• r-imTTnn ti COCQCQCQCQCQCOCQOCOC£>CO<COOC£>C£>CQeOCQCOCOCQCO?£)CQCQOOCOCOCQ
A^ipiaiLLH TiJJCnO', ^^^OGOO'DTiGOOGOGOQTiai^TJ^QGO^JlQOa^Cl CD
oj	—<
• p Wd M9G ro-Jio^G^ocdaooto 'ncoao^DCQoaocor^Gciadr^Qi'^iOi^oo©^'^ co
	CT CT fl! CT CT —I CT CO CT CT CT CO CO CO-H —i	—i CT_COCOCOCOCOCT—ICT CT
-d >S_______________________________________________________fn
'3Jnxdaiax	cd -3 -i id oo cd cd cd cd go id -4 cd o coin o t- t- oo (A oo cd Tf t- id
	CT CT CT CT —I CT CT —I CT CO CO CT CO CO CO CO CT ri -h —i CT	CO CO CO CO CO CT —i CT CT
d________________________________________________________. >>'....	i
,®3g=-£ ^«2p4£i?a2^
o	...............................................................
___________—t-H^COMtCOCOOCOCOCTi-!Tt<—tCTCTCOCTCO^t^COCOCTCTr-i^—t—t—ti-i1
spno[3 jo !,tu y © cd o o o o’o oooo—iinooCTaooaoooaooaoooocbo|
in t" in ci	" oo ©	m	co
.	_________03 CO T	CT	—C	00	Cl CD	Cl
k urea jo UJ _	12 CT	CT	—I	T -c	00
J ___________•	•	•___■__________________________________________ '______CT
I
q	. io cd	oo oo m ci	ocioq ocoooioct©©ct ©ct	—<	ci
■uioreg jo	’oi •	oo oo'oo oo	ci oo ci ci ci ci ci ci ci ci ci ci cd ci oo	ci	oo
ICT CT	CT CT fl! CT	CTCTCTCTCTCTCTCTCTCTCTCTCTCTCT	CT	CT
—i CT co	mcDr-oocio—iCTco'Oiincot—Gocio—<ctco-^< in'co t- oo ci o —<
______________i—(rH-H—1-3—i—iCTfl!CTCTCTCTCTCTCTfl!COCO
				

